# Circular Material Solutions in Building Mortars: Technical Performance, Environmental Impacts and Economic Implications—A Review

**DOI:** 10.3390/ma19214553

**Published:** 2026-10-28

**Authors:** Natalia Gasik-Kowalska, Małgorzata Kacprzak, Jolanta Baran, Wojciech Kubissa

**Affiliations:** 1 Faculty of Civil Engineering, Mechanics and Petrochemistry, Warsaw University of Technology, 00-661 Warsaw, Poland; malgorzata.kacprzak@pw.edu.pl (M.K.); wojciech.kubissa@pw.edu.pl (W.K.); 2 Department of Economics and Informatics, Faculty of Organization and Management, Silesian University of Technology, 41-800 Zabrze, Poland; jolanta.baran@polsl.pl

**Keywords:** circular economy, building mortars, recycled aggregates, supplementary cementitious materials, sustainable construction, life cycle assessment

## Abstract

**Highlights:**

**What are the main findings?**

- Recycled aggregates represent the most extensively investigated and practically implemented circular solution for cement mortars.
- Supplementary cementitious materials reduce clinker consumption and environmental impacts.
- Mortar performance depends on material type, replacement level, and mix composition.

**What are the implications of the main findings?**

- Integrated LCA and LCC assessments are needed to support material selection.
- Harmonized testing methodologies would improve result comparability.
- Future mortars may combine circularity, self-healing, and CO_2_ sequestration technologies.

**Abstract:**

The growing demand for sustainable building materials has accelerated the implementation of circular economy principles in the construction sector. Among cement-based materials, mortars offer significant opportunities for utilizing secondary raw materials and industrial by-products due to their compositional flexibility and wide range of applications. This review discusses the application of circular economy material solutions in cement mortars, including recycled aggregates, complementary cementitious materials, alternative binders, and recycled mixing water. Their impact on fresh properties, mechanical properties, durability, and functional properties, as well as related environmental and economic aspects, is analyzed. The studies reviewed indicate that the performance of mortars containing circular materials depends on the material type, its properties, the level of exchange, and the mix composition. Partial cement substitution commonly results in GWP reductions of approximately 10–40%, whereas higher replacement levels, alternative binder systems, recycled fibres, and carbon-storing materials may provide greater reductions under favourable conditions. Many factors influence the environmental and economic benefits, including processing requirements, transportation distances, local availability of secondary resources, and life-cycle assumptions. Despite significant progress in recent years, challenges related to material variability, standardization, and long-term performance still limit the wider use of circular materials in mortar production. Further research should therefore focus on improving material characterization, harmonizing assessment methods, and supporting the practical implementation of circular solutions in the construction sector.

## 1. Introduction

The construction sector is one of the largest consumers of natural resources and a major source of waste generation and greenhouse gas emissions worldwide. Economic growth and the increasing demand for infrastructure development have intensified the extraction of raw materials and the generation of substantial quantities of construction and demolition waste. Consequently, the construction industry is widely recognized as one of the most resource- and energy-intensive sectors of the global economy [1,2].

The intensive consumption of natural resources and the associated environmental pressures have driven the development of regulatory and technological measures aimed at mitigating the environmental impacts of construction activities [3]. In this context, the transition of the construction sector towards a circular economy has become increasingly important, with the primary objectives of improving resource efficiency and reducing waste generation. The development of innovative construction materials incorporating secondary raw materials and industrial by-products, together with the adoption of sustainable technological solutions, currently represents one of the key pathways supporting this transition [1].

### 1.1. Background

The substantial environmental impact of the construction sector stems from its extensive consumption of resources and intensive waste generation. The industry is estimated to account for approximately 35% of global energy consumption, 30% of greenhouse gas emissions, and 40% of natural resource use [2,4,5]. Moreover, the construction sector generates approximately 40% of the global waste stream, a significant proportion of which originates from the demolition of existing buildings and infrastructure [2,6,7]. Cement production remains one of the most energy-intensive and carbon-emitting industrial processes, contributing substantially to global CO_2_ emissions and representing a major challenge in achieving climate targets [1,2].

Construction mortars play a key role in advancing circularity within the construction sector. Owing to their widespread use and diverse technological functions, mortars provide a suitable platform for the implementation of circular solutions [8,9]. The incorporation of secondary raw materials and industrial by-products into mortar formulations offers significant potential for reducing the consumption of virgin resources while maintaining or enhancing material performance [10]. However, the heterogeneous nature and variability of waste-derived materials present significant challenges to their effective utilization, necessitating a comprehensive evaluation encompassing technological, environmental, and economic aspects [6,8]. This highlights the need for an integrated research approach to the assessment of circular solutions, which remains insufficiently addressed in the current body of literature [8].

### 1.2. Circular Economy in Construction

In the construction sector, these principles are translated into measures aimed at improving resource efficiency and reducing waste generation. This approach is implemented through strategies such as life-cycle extension of materials, increased use of secondary raw materials, and the development of recovery and reuse processes [6,11,12].

The implementation of circular economy principles in construction relies on waste prevention, material recovery, and the use of secondary resources in subsequent production cycles. However, a substantial proportion of construction and demolition waste is still managed through low-value recovery routes, limiting the effectiveness of material loop closure [5,13,14].

Due to its substantial consumption of raw materials and the characteristics of the waste it generates, the construction sector exhibits considerable potential for the implementation of circular solutions. These include selective demolition, the reuse of structural components, design for disassembly, and the integration of recycled materials into new construction applications [15]. Digital tools also play an important role in this transition, particularly Building Information Modeling (BIM), which facilitates optimized material management throughout the entire life cycle of a building [7,16].

Despite the growing relevance of these approaches, their implementation faces numerous challenges, including the lack of standardized procedures, the variable quality of secondary materials, and limited market acceptance [5,17]. Furthermore, a substantial proportion of construction and demolition waste continues to be landfilled or used in low-value applications, indicating the limited effectiveness of current practices in achieving circularity objectives [12].

Consequently, the assessment of circular economy performance increasingly relies on circularity indicators, such as the Circular Material Use Rate (CMUR) and the Material Circularity Indicator, which enable the analysis of material flows and the extent of secondary material utilization [18,19]. However, the literature highlights the need for multidimensional assessment frameworks capable of better capturing the complexity of processes occurring within the construction sector [20]. The absence of consistent and integrated approaches for evaluating the effectiveness of circular solutions further hinders their large-scale implementation and limits the comparability of findings across studies [20,21].

Accordingly, the effective transformation of the construction sector requires the integration of technological, regulatory, and economic considerations within comprehensive circular economy strategies [1,8]. Particular attention should be directed towards the identification of material solutions capable of effectively closing material loops while maintaining the required performance characteristics [9,11]. Nevertheless, current practices remain insufficient to fully achieve this objective [8,20].

### 1.3. Why Mortars?

Among construction materials, mortars represent a particularly relevant area of investigation due to both the scale of their use and their functional significance in construction practice. They are widely employed as bonding materials for structural elements, particularly in masonry construction, as well as in finishing applications such as renders, screeds, and adhesive systems. Consequently, modifications to their material composition can generate substantial environmental benefits at the scale of the entire construction sector [22,23].

A key characteristic of construction mortars is the flexibility of their composition. As composite materials consisting primarily of a binder, fine aggregate, and water [24], often supplemented with various admixtures and additives, mortars provide considerable opportunities for tailoring material properties through the partial replacement of conventional constituents with alternative materials [9]. This is particularly relevant for cement-based mortars, whose production is associated with significant environmental impacts, primarily due to the manufacture of Portland cement and clinker-intensive binders [22].

From the perspective of reducing environmental burdens, the incorporation of waste-derived materials and industrial by-products as partial substitutes for conventional mortar constituents is of particular importance. The use of materials such as ground granulated blast-furnace slag, fly ash, silica fume, and recycled aggregates can reduce the consumption of virgin raw materials [25,26] while simultaneously lowering emissions associated with the production of construction materials [27]. Moreover, these approaches support more efficient waste management practices by enabling the valorization of waste streams in higher-value applications within a circular economy framework [5,16].

The environmental performance of construction mortars is primarily determined by the properties and production processes of their constituent materials, among which the binder typically represents the dominant contributor. In cement-based mortars, the main environmental impacts arise from clinker production, fuel consumption, and the decarbonization of limestone during cement manufacture [28]. Aggregate extraction and material transportation also contribute significantly to the overall environmental footprint of mortar systems [22,29].

Despite their considerable potential for reducing environmental impacts, the incorporation of secondary materials into mortar formulations requires a comprehensive approach that considers durability, performance characteristics, and the potential for further material recovery at the end of service life. Appropriate mix design can contribute to extending service life, reducing maintenance requirements, and minimizing waste generation [22,29,30]. Consequently, construction mortars represent a particularly relevant field of research for the evaluation and implementation of circular solutions, combining widespread application with a high potential for material modification and the efficient utilization of secondary resources, thereby highlighting their important role in the transition towards circular construction systems [1,16].

### 1.4. Aim and Novelty

This review critically evaluates the technical, environmental and economic implications of circular material solutions in building mortars, including recycled aggregates, supplementary cementitious materials, alternative binders and recycled water systems.

Despite the growing number of studies on circular materials in construction, existing research is often fragmented and primarily focused on either technical performance or environmental aspects, with limited integration of economic considerations [1,8]. Moreover, the majority of available reviews address concrete systems, while less attention has been given to building mortars, despite their widespread use and high potential for material substitution [9,22].

Therefore, this paper provides a comprehensive and integrated assessment of circular material solutions in mortars, combining an analysis of mechanical performance, durability, environmental impacts, and economic feasibility. The novelty of this review lies in bringing together technical, environmental, and economic perspectives on circular material solutions in building mortars within a single analytical framework. By synthesizing evidence across these dimensions, the review identifies key knowledge gaps, research priorities, and opportunities for the broader implementation of circular mortar systems.

## 2. Methodology

A structured literature review methodology was adopted to comprehensively evaluate circular material solutions in building mortars. The analysis focused on the identification and critical assessment of studies addressing the technical, environmental, and economic dimensions of circular mortar systems. The literature search strategy, study selection procedure, and data analysis approach are described in the following sections.

### 2.1. Literature Search Strategy

The literature search was conducted using the Scopus and Web of Science databases between 11 May and 18 June 2026. Publications published between 2010 and 2026 were considered to capture recent developments in circular material solutions for mortar systems. The year 2010 was selected as the starting point because research on circular construction materials and resource-efficient technologies began to gain significant momentum during this period.

The search strategy was developed to identify studies addressing the technical performance, environmental impacts, and economic implications of circular materials used in mortars. Multiple combinations of keywords were used to identify studies relevant to the scope of this review. The search strategy incorporated keywords related to mortar systems (e.g., mortar, masonry mortar), circular economy concepts (e.g., circular economy, recycling, sustainability, environmental impact, life cycle assessment, carbon footprint, life cycle costing), and specific material groups (e.g., recycled aggregate, fly ash, slag, supplementary cementitious materials, alkali-activated materials, geopolymer, recycled water, PET fibre, and industrial waste).

Searches were performed within article titles, abstracts, and keywords. Only peer-reviewed journal articles published in English between 2010 and 2026 were considered. Additional references were included where necessary to provide contextual background and support the discussion of specific topics. Selected elements of the PRISMA framework [31] were adopted solely to enhance the transparency of the literature identification and study selection process; however, the review was not conducted in full accordance with PRISMA guidelines and was not intended as a formal systematic review.

### 2.2. Search Keywords

The search strategy was based on combinations of keywords corresponding to the main thematic areas addressed in this review. The keyword groups used during the literature search are presented in Table 1.

### 2.3. Selection Procedure

After removing duplicate records, titles and abstracts were screened to identify studies relevant to the scope of this review. Publications meeting the initial screening criteria were subsequently subjected to full-text assessment. Studies that did not satisfy the predefined inclusion criteria were excluded at successive stages of the selection process.

**Figure 1:**
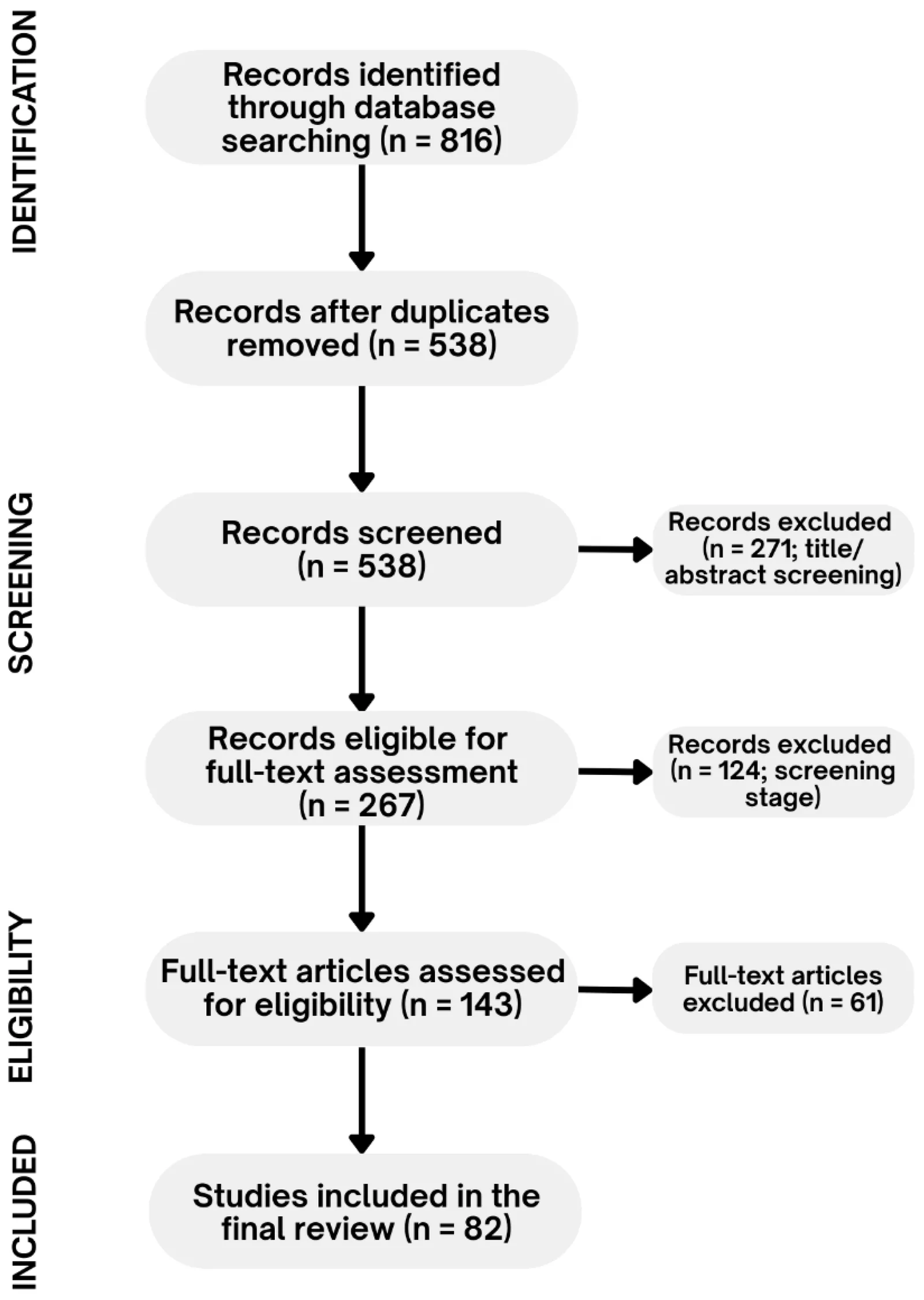
Flow diagram illustrating the literature search and study selection process.

A detailed overview of the study identification, screening, eligibility assessment, and inclusion process is presented in Figure 1.

Inclusion criteria comprised peer-reviewed journal articles published in English between 2010 and 2026 that addressed circular materials in building mortars and reported technical, environmental, or economic outcomes. Conference papers, editorials, theses, and studies not directly related to mortar systems were excluded.

### 2.4. Study Quality Assessment

To ensure the reliability and relevance of the reviewed literature, an additional quality assessment was conducted prior to data synthesis. The assessment considered several criteria, including relevance to circular material solutions in mortar systems, clarity and reproducibility of the experimental methodology, adequacy of material characterization, completeness of reported technical, environmental, or economic results, and consistency between objectives, methods, and conclusions.

Studies lacking sufficient methodological information, presenting incomplete datasets, or providing only limited discussion of mortar performance were excluded from the final synthesis. Particular preference was given to studies reporting quantitative data related to fresh-state properties, mechanical performance, durability, life-cycle assessment (LCA), resource efficiency, and life-cycle costing (LCC).

Following the quality assessment, the selected studies were classified according to the type of circular material investigated, including recycled aggregates, supplementary cementitious materials, alternative binders, recycled water systems, recycled fibres, and recycled admixtures. This classification formed the basis of the thematic synthesis presented in the subsequent sections.

### 2.5. Data Extraction and Synthesis

Whenever available, the following information was collected: material type, replacement level, mortar composition, fresh-state properties, mechanical performance, durability characteristics, environmental indicators, and economic aspects. Particular attention was given to parameters frequently reported in the literature, including compressive strength, flexural strength, workability, freeze–thaw resistance, chloride penetration, carbon footprint, resource efficiency, and Life Cycle Costing (LCC).

Due to the significant heterogeneity of the materials studied, experimental procedures, replacement levels, and performance indicators reported in the literature, a quantitative meta-analysis was not deemed appropriate. Instead, the collected data was synthesized using a thematic approach, which allowed for the identification of common trends and differences between material groups.

## 3. Circular Material Solutions in Mortars

Materials used in building mortars can be classified according to both their technological function and origin [22,32]. From a functional perspective, four main groups of constituents can be distinguished: binders, aggregates, mixing water, and additives/admixtures, which collectively govern the properties of the fresh mix and the performance of the hardened material [22,32].

From a circular economy perspective, the classification of materials according to their origin is particularly relevant, encompassing primary raw materials, secondary materials derived from recycling processes, and industrial by-products [1,33]. Primary materials, including natural aggregates, conventional binders, and water, form the basis of mortar production; however, their extraction and processing are associated with considerable environmental burdens [22,28]. In response to these challenges, increasing attention has been directed towards the utilization of secondary materials and industrial by-products, which can reduce the consumption of virgin resources while promoting the valorization of waste streams [1].

**Figure 2:**
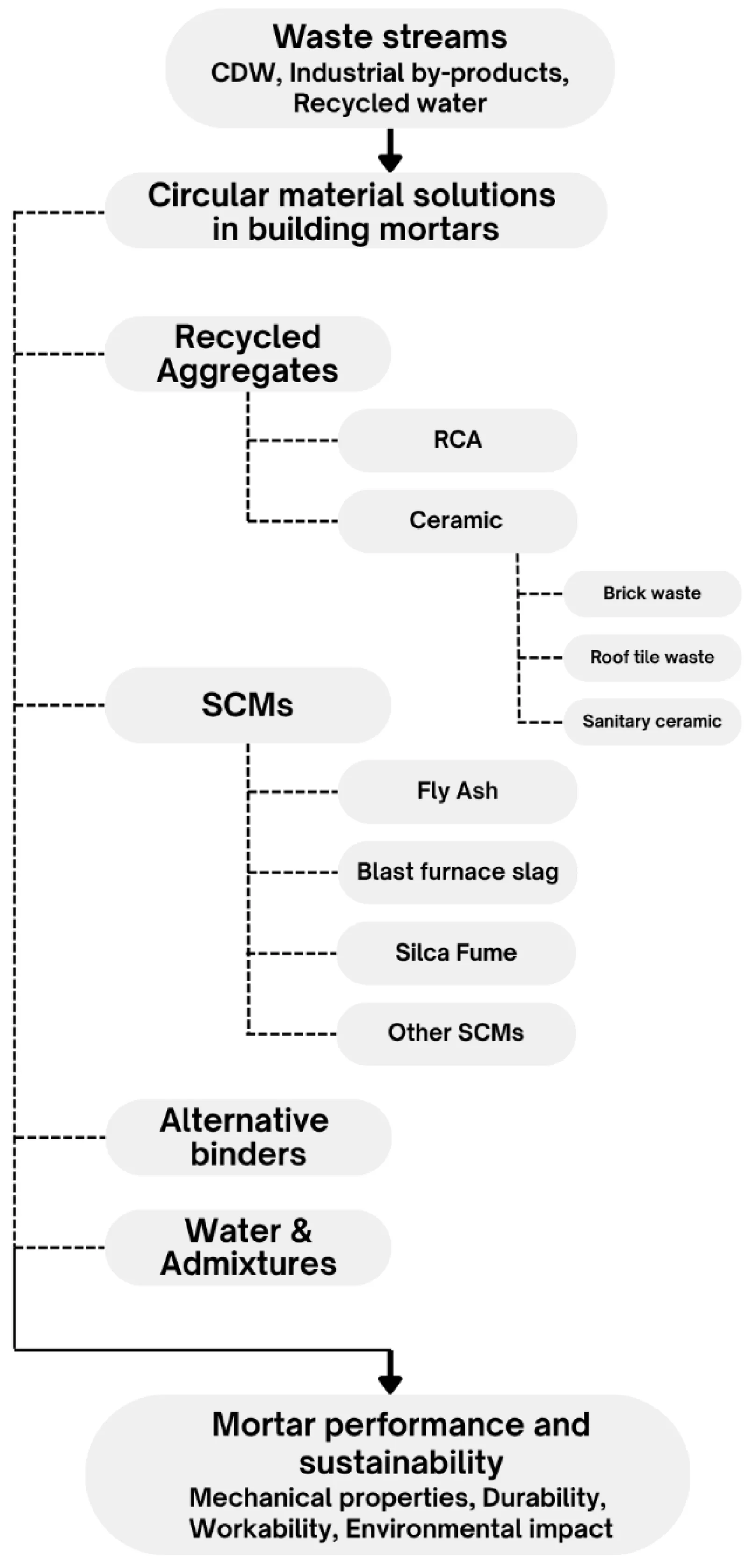
Conceptual framework of circular material solutions in building mortars, illustrating the flow from waste streams to material incorporation and their resulting effects on mechanical performance, durability, workability, and environmental impact.

This section presents the main categories of circular material solutions applied in building mortars and discusses their role within the respective constituent groups. The section is structured according to the functional classification of mortar components, providing a systematic framework for analyzing their contribution to mortar performance and circularity. The relationships between the different material categories and their potential applications are illustrated in Figure 2.

### 3.1. Recycled Aggregates

Mineral aggregates are among the key constituents of building mortars, serving as the filler phase and significantly influencing the microstructure, mechanical properties, and dimensional stability of the material [34]. Fine aggregates, particularly natural sand, play a dominant role in mortar formulations, as their gradation and physical characteristics directly affect workability, density, and strength-related properties of the composite [34,35].

Within a circular economy framework, an important strategy is the partial or complete replacement of natural aggregates with recycled materials. Recycled aggregates are primarily derived from construction and demolition waste (CDW), particularly recycled concrete aggregate (RCA) and ceramic waste originating from bricks and roof tiles [33,35,36]. Recycled sanitary ceramic materials are also gaining increasing attention as an alternative source of mineral constituents for mortar production [37,38].

However, the use of recycled aggregates is associated with physicochemical characteristics that differ from those of natural aggregates. Higher porosity and water absorption, as well as the presence of adhered cement paste in RCA or glaze residues in ceramic materials, may increase water demand and alter the rheological behaviour of fresh mixtures [35,39,40]. Consequently, the mechanical performance and durability of mortars are strongly influenced by the type of recycled material, its processing route, and the level of aggregate replacement [35,41]. The main advantages and limitations of different types of recycled aggregates are summarized in Table 2, allowing a direct comparison of their application potential and associated constraints.

An additional challenge is the lack of standardized guidelines for the design of mortars incorporating recycled aggregates, which often necessitates an experimental approach and mixture-specific optimization [42]. Despite these limitations, recycled aggregates represent one of the most promising pathways for implementing circular economy principles in the construction sector. Their widespread availability, extensive research background, and practical applicability have contributed to their increasing adoption in mortar production [35,40,42]. When properly incorporated into mortar formulations, they can reduce the consumption of virgin resources and divert waste from disposal streams while maintaining the required performance characteristics [7,12,40].

Nevertheless, their performance remains highly dependent on raw material quality and absorption properties. Challenges related to increased water demand, mechanical performance, and durability still require careful optimization of both processing procedures and mix design [39,40,41].

### 3.2. Supplementary Cementitious Materials (SCMs)

Binders play a particularly important role in building mortars, as they govern the processes of setting and hardening and are largely responsible for the long-term durability of the material [22,43]. At the same time, binders represent the most energy-intensive component of mortar formulations and constitute the primary source of carbon dioxide emissions associated with their production [28]. Consequently, binder design has become a key area for reducing the environmental impacts of construction materials, including the incorporation of secondary materials and industrial by-products into binder systems [32]. In parallel, optimizing binder composition while maintaining the required durability can contribute to lower consumption of virgin resources and reduced waste generation throughout the material life cycle [27,29].

Portland cement remains the most widely used binder in building mortars; however, its production is associated with substantial environmental burdens and represents one of the major sources of CO_2_ emissions within the construction sector [22,28]. These emissions originate primarily from the energy-intensive clinker production process and the decarbonization of calcareous raw materials during cement manufacture [28,44]. Efforts to reduce the environmental footprint of cement-based materials are therefore largely focused on decreasing clinker content through the partial replacement of cement with supplementary cementitious materials (SCMs) possessing pozzolanic or hydraulic properties [27,32].

The most commonly used SCMs include fly ash (V), blast furnace slag (S), silica fume (D), biomass ashes, and finely ground powders derived from construction and demolition waste (CDW) [29,32]. Their typical cement replacement levels and effects on the mechanical performance and durability of cementitious composites are summarized in Table 3. As a by-product of coal combustion, fly ash exhibits pozzolanic activity, reacting with calcium hydroxide to form secondary hydration products that enhance the density and durability of cementitious matrices [26,32]. Blast furnace slag, characterized by latent hydraulic properties, contributes to reduced permeability and improved chemical resistance [32,45]. Due to its extremely fine particle size and high content of reactive silica, silica fume promotes hydration reactions and refines the microstructure, particularly within the interfacial transition zone (ITZ) between aggregate particles and the cement paste, which plays a crucial role in determining the mechanical performance of cementitious composites [32,46].

For biomass ashes, material performance is strongly influenced by the type of biomass used and combustion conditions. Depending on their composition, these materials may exhibit pozzolanic activity or act as microfillers, contributing to matrix densification and modifying the rheological properties of fresh mixtures [10,32,47]. Similarly, powders derived from construction and demolition waste (CDW), such as finely ground concrete or ceramic materials, exhibit variable reactivity but can function as either supplementary cementitious materials or microfillers. In both cases, they may improve particle packing and reduce the porosity of the cementitious matrix [33,34,48].

The incorporation of SCMs therefore not only reduces emissions associated with clinker production but also enables the modification of microstructure and durability characteristics. As a result, SCMs represent a key component in the design of modern low-carbon cement-based materials [28,29,32].

Compared to recycled aggregates, cementitious supplementary materials offer greater potential for reducing the environmental footprint of mortars by replacing clinker. However, their long-term availability is likely to decline with the phasing out of coal-fired energy, underscoring the need to find alternative cementitious supplementary materials.

### 3.3. Alternative Binders

In response to increasing environmental requirements, growing attention has been directed towards alternative mineral binders that align with circular economy principles and decarbonization strategies within the construction sector [28,29]. Among the most actively investigated solutions are alkali-activated materials (AAMs), including geopolymers, which are produced from secondary raw materials such as fly ash and blast furnace slag. These materials enable a substantial reduction in clinker consumption and the associated CO_2_ emissions while maintaining favorable mechanical properties and durability [32,50]. Their formation is based on the dissolution and reprecipitation of aluminosilicate phases in an alkaline environment, leading to the development of amorphous or poorly crystalline binding products with a gel-like structure [50].

At the same time, the importance of low-carbon cements is increasing, with reduced clinker content achieved through the incorporation of mineral additives possessing pozzolanic or hydraulic properties [28,32]. A particularly important group comprises blended cements and Limestone Calcined Clay Cement (LC3) systems, in which the clinker content can be reduced to approximately 35% due to the synergistic interaction between calcined clay and limestone [51,52]. In these materials, stable phases such as carboaluminates are formed, contributing to improved microstructure and durability [51]. In addition, materials such as siliceous fly ash, blast furnace slag, and fine fractions derived from recycled concrete are utilized [49], promoting more efficient resource use and reducing the consumption of virgin raw materials [1,29].

In addition to the most extensively studied systems, such as AAMs and low-carbon cements, other groups of binders with a reduced environmental footprint are also being actively developed, including calcium sulfoaluminate (CSA) cements and magnesia-based binders [28,53,54]. CSA cements are characterized by lower firing temperatures, reduced CO_2_ emissions, and rapid strength development resulting from intensive ettringite formation [55]. Magnesia-based binders, which are based on magnesium oxide or magnesium carbonates, can absorb CO_2_ during hardening through carbonation processes, further enhancing their emission-reduction potential [54,56]. These technologies enable reductions in production energy demand, clinker content, and the effective utilization of industrial by-products, representing an important direction for the development of sustainable construction materials [32].

The literature has also reported the application of other secondary resources, including quarry waste [57], agricultural ashes [58], recycled brick waste [59], and waste-derived mineral fillers [60], further illustrating the growing diversity of circular material solutions investigated for mortar production.

Alternative binder systems represent one of the most promising avenues for reducing the environmental impact of mortar production. However, despite their significant environmental potential, their wider implementation is still limited by technological complexity, standardization challenges, and the limited availability of long-term performance data. These factors continue to hinder their large-scale adoption despite the considerable environmental benefits they may offer.

### 3.4. Recycled Water and Chemical Admixtures

Water is a fundamental and indispensable component of building mortars, governing the hydration of the binder, the rheological properties of the mixture, and the initiation of setting and hardening processes [43,61]. From an environmental perspective, its consumption is associated with significant pressure on natural resources, particularly in the context of limited availability of surface and groundwater resources. This justifies the need to reduce the use of freshwater through the implementation of water recovery and reuse strategies in technological processes [62,63].

In industrial practice, closed-loop water systems are increasingly employed, whereby water used in auxiliary operations, such as equipment cleaning and material transport, undergoes secondary treatment processes including sedimentation, filtration, and chemical conditioning [64]. The resulting process water can be reused in mortar production, thereby reducing freshwater consumption and decreasing the generation of industrial wastewater, which represents a significant environmental burden [65]. At the same time, the use of recycled water requires careful control of water quality parameters, particularly the concentration of dissolved ions, organic contaminants, and suspended solids, all of which may affect hydration processes and the final properties of cementitious composites [63,66]. In this context, water quality monitoring systems play an important role by enabling the assessment of water suitability for reuse in accordance with relevant standards and technical requirements [67,68].

Alongside efforts to optimize water management, increasing attention has been directed towards components that modify mortar properties, including chemical admixtures and additives. Their use enables the adjustment of rheological behaviour, regulation of setting time, and improvement of durability and mechanical performance without increasing the clinker content or mixing water demand [69,70]. In particular, superplasticizers and plasticizers allow for a reduction in the water-to-binder ratio, contributing to improved microstructure and enhanced durability of mortars [69].

Within the framework of the circular economy, growing interest has also been directed towards recycled admixtures and additives, including products manufactured from industrial by-products and recovered chemical resources [1,28]. This group includes modified superplasticizers, mineral–polymer additives [69,71], as well as components derived from the processing of industrial and construction waste streams [32,72]. Their application contributes to reducing the consumption of primary chemical resources and lowering the environmental footprint of construction materials [28,29]. However, ensuring compositional stability and consistent performance under industrial conditions remains a significant challenge [32,73].

An important area of development also involves recycled polymer-based additives, particularly synthetic fibers obtained from plastic waste such as polyethylene terephthalate (PET) [74,75]. When used as dispersed reinforcement in mortars and cement-based composites, PET fibers improve crack resistance, increase ductility, and enhance resistance to dynamic loading [75]. Their utilization also supports circular economy principles by enabling the valorization of waste streams that are difficult to recycle in other sectors [74,75]. Despite these advantages, their wider implementation is limited by issues related to fiber–matrix adhesion and the variability of properties associated with secondary raw materials [73].

Complementary solutions include bio-based additives such as natural fibers, biopolymers, and starch-based modifiers, which are often derived from agricultural and industrial waste streams [76,77]. These materials can improve rheological behaviour and selected mechanical properties of mortars while reducing their environmental impacts [73,77]. However, ensuring long-term durability and resistance to degradation under service conditions remains a major challenge, owing to their susceptibility to biodegradation, microstructural changes, and limited stability in alkaline environments [47,73].

**Figure 3:**
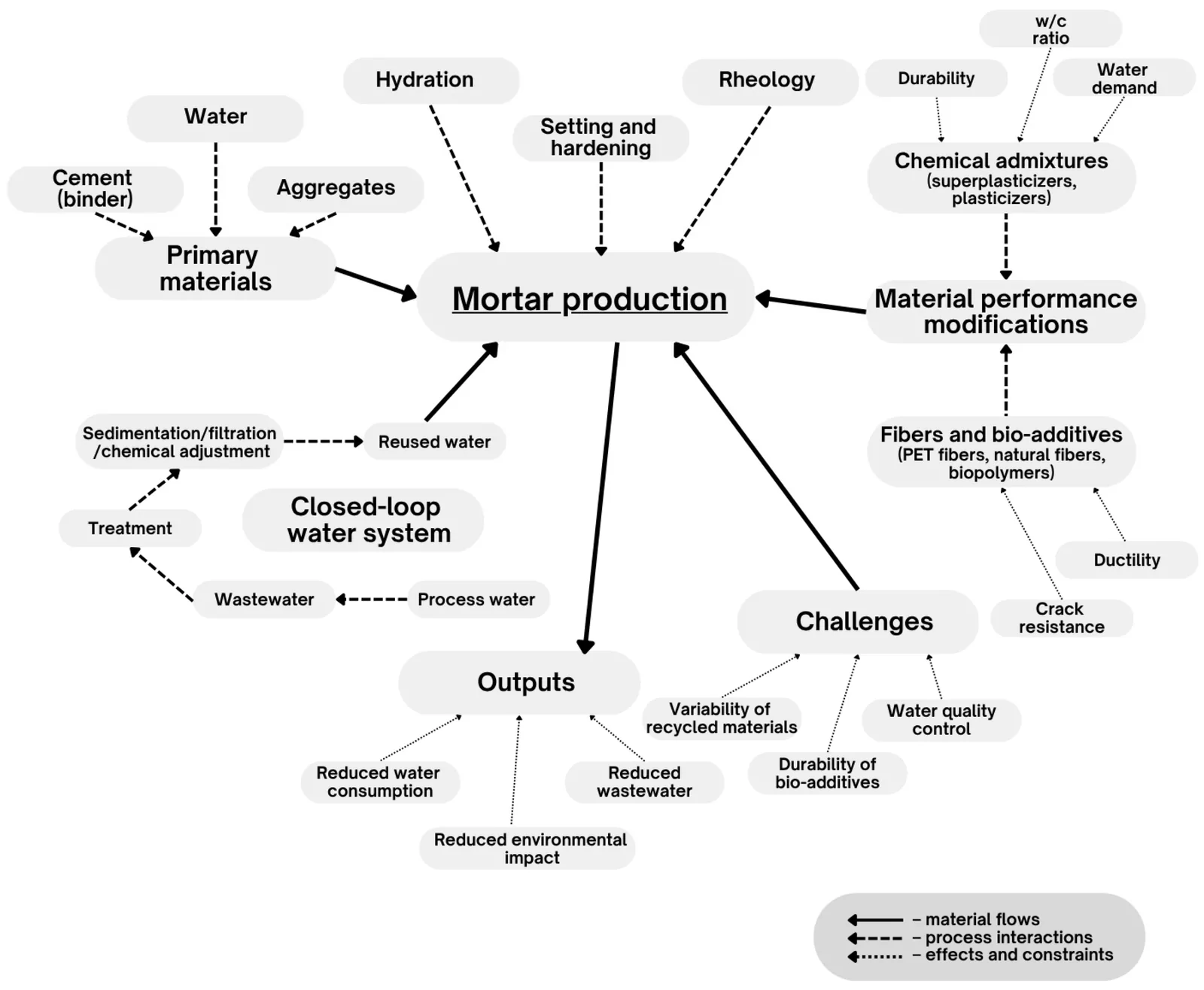
Material flows in circular mortar production, showing the integration of primary raw materials, recycled inputs, and chemical and bio-based modifiers, together with a closed-loop water system and key environmental benefits and limitations.

The integration of recycled process water, low-impact chemical admixtures, and polymeric and bio-based additives represents one of the key directions in the development of modern building mortars [64,69,73], as illustrated in Figure 3. These approaches contribute to improving resource efficiency, reducing emissions, and optimizing material utilization, while supporting long-term sustainability strategies within the construction sector [1,28,29].

## 4. Influence on Technical Performance

Secondary materials significantly influence the technical and performance properties of building mortars. Their effects are complex and depend on the type of material, its microstructure, reactivity, and proportion within the mixture, affecting both the properties of fresh mortars and the performance of the hardened material [9].

The incorporation of recycled materials leads to modifications in the microstructure of cementitious composites, including porosity, the interfacial transition zone, and hydration processes, resulting in changes in rheological behaviour, mechanical properties, durability, and functional characteristics of mortars [34,46].

These effects are highly dependent on the characteristics of the incorporated materials, particularly fine powders and recycled aggregates. The variability of secondary raw materials represents a significant design challenge, requiring appropriate mixture optimization [9,35].

**Figure 4:**
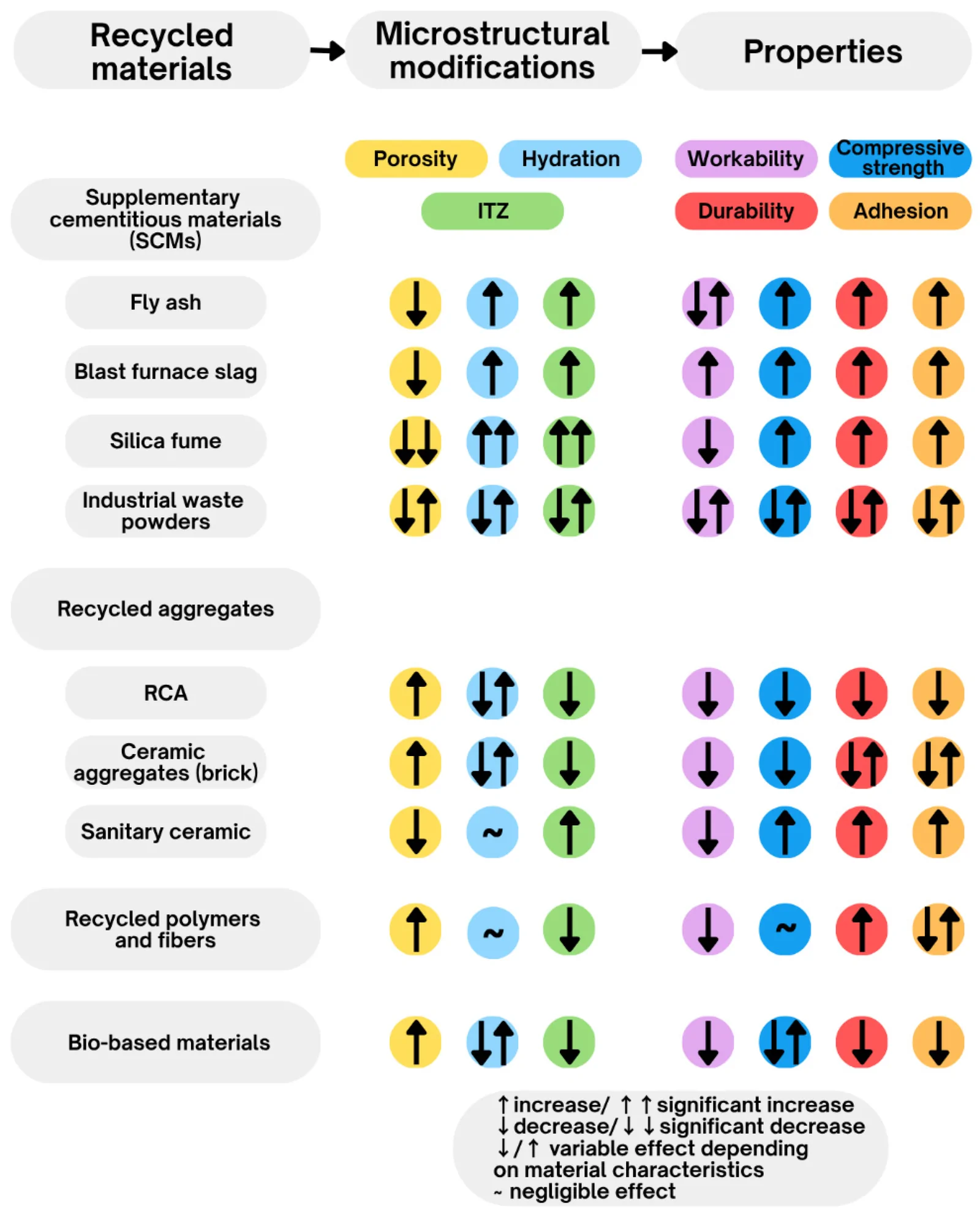
Conceptual synthesis of the main relationships between recycled materials, microstructural modifications (porosity, hydration processes and the interfacial transition zone, ITZ), and selected key mortar properties, including workability, compressive strength, durability, and adhesion (authors’ elaboration based on the reviewed literature).

The relationships between the type of secondary materials, microstructural modifications, and mortar properties are illustrated in Figure 4. Accordingly, the following sections examine fresh-state properties, mechanical performance, durability, and functional properties of mortars.

### 4.1. Fresh-State Properties

The fresh-state properties of mortars influence the technological processes associated with their mixing, transport, and application. Among these properties, workability, water demand, and setting time are of particular importance, as they are strongly influenced by the type and characteristics of the secondary materials incorporated into the mixture [10,34]. Changes in these parameters primarily result from differences in particle size distribution, specific surface area, porosity, and material reactivity, which affect hydration processes and the rheological behaviour of cement-based mixtures [46,61].

#### 4.1.1. Workability

For supplementary cementitious materials (SCMs), the influence on workability depends primarily on particle morphology and specific surface area. Fly ash often improves rheological properties due to the spherical shape of its particles; however, the observed effect depends on its composition, fineness, and cement replacement level [26]. Similar trends have been reported for blast furnace slag, which generally does not impair workability and, in many cases, may contribute to its improvement [32,78]. In contrast, silica fume, characterized by a very high specific surface area, increases water demand, raises mixture viscosity, and reduces workability [46]. The effect of powders derived from industrial waste is less consistent and depends on their chemical composition, particle size distribution, and porosity. Materials acting primarily as fillers may improve particle packing and enhance workability, whereas more porous fractions increase water demand and adversely affect rheological properties [25,46].

Significant changes in workability have also been observed in mortars incorporating recycled aggregates. Both recycled concrete aggregates (RCA) and ceramic aggregates derived from masonry demolition waste are typically characterized by high water absorption and a porous microstructure, resulting in increased water demand and reduced workability and plasticity of mortar mixtures [34,35]. More favorable performance has been reported for recycled sanitary ceramic aggregates, which generally exhibit lower porosity and higher density, leading to a less pronounced effect on the rheological properties of mortars [38,41,79].

The incorporation of recycled fibers and polymers generally leads to reduced workability of fresh mortars due to increased internal friction and restricted flowability of the mixture [73,75]. Similar effects have been reported for bio-based additives, such as natural fibers and biopolymers, whose water absorption capacity contributes to increased viscosity and higher water demand of the system [76,77].

In general, cementitious additives exhibit the most variable impact on workability due to differences in particle morphology and specific surface area. Recycled aggregates, on the other hand, consistently increase water requirements due to their porous structure, while recycled fibers and bio-based additives reduce workability primarily through increased internal friction. Therefore, mix optimization remains crucial to achieving satisfactory green properties after the addition of recycled materials.

#### 4.1.2. Water Demand

Water demand is one of the key design parameters of mortars incorporating secondary materials and is primarily influenced by the specific surface area, porosity, and water absorption capacity of the materials used [45,61]. Among supplementary cementitious materials (SCMs), the effect on water demand varies considerably. Fly ash may reduce water demand due to its favorable particle morphology, whereas silica fume typically increases it because of its high specific surface area and reactivity [26,46]. In the case of waste-derived powders, the observed effect depends on their composition and degree of fineness, with more porous fractions exhibiting a greater capacity for water absorption [25,61].

The most pronounced increase in water demand is generally associated with recycled aggregates and bio-based materials, owing to their porous structure and high water absorption capacity [35,76,77]. Similar tendencies may also be observed when recycled fibers and polymers are incorporated, particularly at higher replacement levels or dosages within the mixture [73,75].

#### 4.1.3. Setting Time

The influence of secondary materials on setting time varies and depends primarily on their chemical activity. Fly ash often increases setting time because of the slower progression of pozzolanic reactions, whereas blast furnace slag generally exhibits a more moderate effect on this parameter [26,32]. In contrast, silica fume may accelerate microstructural development and promote faster densification of the cementitious matrix, which can result in a shorter setting time [46].

Mineral-based recycled materials, such as recycled concrete aggregates, ceramic aggregates, and waste-derived industrial powders, generally exert a less pronounced influence on setting time. The observed changes are mainly associated with modifications in the water-to-binder ratio and hydration processes [10,34,35]. In the case of bio-based additives, the effect is more complex and depends on their water absorption capacity and the interactions between organic constituents and the cementitious matrix [77,80].

### 4.2. Mechanical Properties

Mechanical properties are among the most important parameters determining the durability and serviceability of building mortars [22,67]. Their development depends on the ability of secondary materials to modify the microstructure of cementitious composites, particularly the degree of matrix densification, the quality of the interfacial transition zone, and the efficiency of stress transfer between individual material constituents [46,81]. Consequently, the effects of recycled materials on strength depend strongly on material characteristics and replacement levels [34,35,81].

#### 4.2.1. Compressive Strength

Supplementary cementitious materials (SCMs) are widely recognized as one of the most effective groups of secondary materials for improving the compressive strength of mortars. Fly ash and blast furnace slag promote strength development, particularly at later curing ages, through the formation of additional hydration products, whereas silica fume typically exhibits the strongest strengthening effect owing to its high pozzolanic activity and ability to densify the cementitious matrix [26,45,46,78,82].

Waste-derived powders exhibit more variable effects, depending primarily on their reactivity and physical characteristics. Reactive materials may contribute to strength enhancement through their participation in the formation of additional hydration products, whereas components with limited chemical activity or high porosity often result in reduced load-bearing capacity of the composite [10,25,45,46].

In the case of recycled aggregates, a reduction in compressive strength is commonly observed with increasing replacement levels. This effect is particularly evident for recycled concrete aggregates (RCA) and ceramic aggregates derived from masonry demolition waste, whose high porosity and the presence of adhered mortar promote the development of local stress concentrations [34,35,40]. More favorable results have been reported for recycled sanitary ceramic aggregates, which, owing to their higher density and lower water absorption, may provide mechanical properties comparable to those of natural aggregates [41,79].

The influence of recycled fibers and polymers on compressive strength is generally limited and depends on their content and distribution within the matrix. Most studies report only minor changes in this parameter, indicating that their primary function is not associated with improving compressive load-bearing capacity [71,74,75]. In contrast, bio-based additives most commonly lead to a reduction in compressive strength due to increased porosity and limited compatibility with the cementitious matrix [76,77].

#### 4.2.2. Flexural Strength

The influence of secondary materials on flexural strength is more complex than in the case of compressive strength, as it depends not only on the degree of matrix densification but also on the ability of the material to limit the initiation and propagation of microcracks. Supplementary cementitious materials (SCMs), including fly ash, blast furnace slag, and silica fume, generally contribute to improved flexural strength through microstructural refinement and enhancement of the interfacial transition zone [26,45,46,78,82].

The effect of powders derived from industrial waste is more variable and depends on their reactivity and degree of integration with the cementitious matrix [10,45,46]. Recycled aggregates most commonly lead to a reduction in flexural strength as their proportion in the mortar increases, although this effect is generally less pronounced in the case of recycled sanitary ceramic aggregates [35,40,41,79].

The most beneficial effects on flexural performance have been reported for recycled fibers, including PET fibers, which enhance the ability of the material to transfer stresses after cracking and limit crack propagation [74,75]. Similar mechanisms have been observed for selected bio-based materials; however, their effectiveness strongly depends on fiber durability and the quality of the bond with the cementitious matrix [76,77].

#### 4.2.3. Adhesion

The adhesion of mortars to substrates depends on the characteristics of the interfacial zone, material porosity, water retention capacity, and the development of the cementitious matrix microstructure [34,45,46]. Supplementary cementitious materials (SCMs), particularly silica fume, fly ash, and blast furnace slag, generally have a positive effect on adhesion due to the densification of the cement paste structure, improvement of the interfacial transition zone, and reduction of microstructural defects [26,45,46,78].

In the case of waste-derived powders, the effect on adhesion depends on their physicochemical properties and their ability to act as reactive or filler materials. Fine-grained materials may improve particle packing and matrix density, whereas additives with high porosity or limited chemical activity may reduce the quality of the bond with the substrate [10,25,45].

Recycled aggregates affect adhesion in a less straightforward manner. The high porosity and water absorption of recycled concrete and ceramic aggregates may weaken the interfacial zone; however, the rough and developed surface texture of these materials can simultaneously promote mechanical interlocking of the mortar with the substrate [35,40]. For recycled sanitary ceramic aggregates, the observed effect depends on the proportion of glazed surfaces and the degree of surface texture development of the particles [38,41].

Recycled fibers and polymers generally influence adhesion indirectly by limiting the development of shrinkage cracks and improving material integrity within the bond zone [73,75,83]. In contrast, bio-based materials may exhibit less stable adhesion performance due to their variable structure, high water absorption capacity, and susceptibility to degradation in the alkaline cementitious environment [76,77,84].

### 4.3. Durability

Durability is one of the key parameters used to evaluate mortars containing secondary materials, as it determines their ability to maintain the required properties under service conditions. The influence of recycled materials on durability varies and depends on both the characteristics of the incorporated components and the type of environmental exposure to which the material is subjected [34,35]. Particular importance is given to processes related to moisture transport, exposure to aggressive chemical substances, and cyclic temperature variations, all of which may lead to the gradual degradation of the mortar structure [34,67].

#### 4.3.1. Freeze–Thaw Resistance

Freeze–thaw resistance is closely related to the ability of a material to limit water absorption and transport, as well as to accommodate the stresses generated during water freezing [34,67]. Supplementary cementitious materials (SCMs), including fly ash, blast furnace slag, and silica fume, generally improve freeze–thaw resistance by densifying the mortar structure and reducing water penetration [45,46,78].

The effect of powders derived from industrial waste is more variable and depends on their physical characteristics and degree of integration with the cementitious matrix [10,45,72]. In the case of recycled aggregates, particularly recycled concrete aggregates (RCA) and ceramic aggregates derived from masonry materials, high water absorption may increase susceptibility to freeze–thaw damage [34,35]. Compared with many ceramic aggregates derived from masonry waste, recycled sanitary ceramic aggregates generally exhibit more favorable freeze–thaw performance owing to their lower porosity and greater resistance to moisture exposure [38,41,79].

The influence of recycled fibers and polymers is largely associated with their ability to limit crack development, which may reduce material degradation during freeze–thaw cycling [73,75,83]. In contrast, bio-based materials may exhibit lower durability under cyclic wetting and freezing conditions due to their greater susceptibility to biological degradation, moisture absorption, and property variations in alkaline environments [76,77,84].

#### 4.3.2. Sulfate Resistance

Sulfate resistance is an important parameter for mortars exposed to chemically aggressive environments. The most favorable results are generally observed for pozzolanic and hydraulic materials, such as fly ash, blast furnace slag, and silica fume, which reduce material permeability, limit sulfate ion transport, and decrease the amount of hydration products susceptible to the formation of expansive phases, such as gypsum and ettringite [26,45,78].

The influence of waste-derived powders depends on their chemical composition and reactivity [10,45,46]. Recycled aggregates may exhibit increased susceptibility to sulfate degradation when their high porosity facilitates the transport of aggressive ions into the material [34,35,81]. For recycled fibers and polymers, the effect on sulfate resistance is generally indirect and is primarily associated with limiting crack development and maintaining the structural integrity of the material [73,75,83]. In contrast, bio-based materials may exhibit greater susceptibility to degradation in chemically aggressive environments, which can limit their long-term durability [76,77,84].

#### 4.3.3. Chloride Penetration

Resistance to chloride ion penetration is one of the most important durability parameters, particularly for structures exposed to de-icing salts or marine environments. Cementitious materials with pozzolanic activity, especially silica fume, fly ash, and blast furnace slag, generally reduce chloride ingress by densifying the microstructure and decreasing the continuity of capillary pores [26,45,78].

The effect of waste-derived powders is more variable and depends on their influence on the compactness of the composite [10,25,45]. In the case of recycled aggregates, higher porosity may facilitate chloride ion transport, whereas recycled sanitary ceramic aggregates generally exhibit more favorable performance in this respect [34,35,41]. Recycled fibers may indirectly reduce chloride ingress by limiting the width and development of cracks within the material [74,75,83].

#### 4.3.4. Carbonation

Carbonation is a natural aging process in cement-based materials, the intensity of which depends on mortar permeability and service conditions. The influence of supplementary cementitious materials (SCMs) on carbonation is not unequivocal and depends on the balance between microstructural densification and changes in the composition of hydration products. Despite improving the compactness of the microstructure, the reduction in portlandite content resulting from pozzolanic reactions may lead to greater carbonation depths when the mixture composition is not appropriately designed [26,45,78].

For waste-derived powders and recycled aggregates, the influence on carbonation is primarily governed by material porosity and the ability of the composite to transport carbon dioxide [34,35,41]. Materials with a denser structure generally exhibit greater resistance to carbonation. Recycled fibers and polymers mainly affect carbonation indirectly through the control of crack development [71,73,75,83], whereas the influence of bio-based materials depends largely on changes in the pore structure of the composite and their long-term stability under service conditions [76,77].

### 4.4. Functional Properties

The incorporation of secondary materials enables the modification not only of the technological and mechanical properties of mortars but also of their functional characteristics. Depending on the type of material used, it is possible to tailor thermal insulation performance, acoustic properties, and advanced functionalities related to energy storage and self-healing capabilities [85,86,87]. These properties are of particular importance in the context of sustainable construction and the development of materials with enhanced energy efficiency [16,86].

#### 4.4.1. Thermal and Acoustic Performance

The thermal and acoustic properties of mortars are strongly influenced by the type of secondary materials used and their effect on the density and pore structure of the material. In the case of supplementary cementitious materials (SCMs) and powders derived from industrial waste, their influence on these properties is generally indirect and mainly associated with changes in the microstructure of the composite [45,46,85].

A particularly pronounced effect on thermal and acoustic performance is observed for recycled aggregates. Recycled concrete aggregates (RCA) and ceramic aggregates derived from masonry materials, owing to their porous structure, may contribute to a reduction in thermal conductivity and improved sound attenuation through increased dissipation of acoustic energy [34,35,88]. Recycled sanitary ceramic aggregates generally exhibit a less pronounced effect on thermal and acoustic properties than the more porous aggregates obtained from red ceramic waste [37,38,41].

Recycled fibers and polymers may further enhance the ability of the material to damp vibrations and limit the development of microcracks, which can positively affect the acoustic performance of the composite [71,73,75]. Beneficial effects have also been reported for bio-based materials, whose porous structure contributes both to reduced heat transfer and increased absorption of acoustic energy [85,88].

As a result, the incorporation of secondary materials enables the development of mortars intended for energy-efficient applications and environments requiring improved acoustic comfort [85,86]. However, increased material porosity may also lead to a reduction in the mechanical strength of mortars [34,35,88].

#### 4.4.2. PCM Systems

Growing interest in energy-efficient construction has contributed to the development of mortars incorporating phase change materials (PCMs). These systems enable the storage and release of thermal energy during phase transitions, thereby reducing temperature fluctuations within buildings [86].

Secondary materials can facilitate the integration of PCMs into the cementitious matrix and influence the efficiency of energy storage. Particularly beneficial in this regard are porous recycled mineral materials, including recycled aggregates, which can act as PCM carriers by immobilizing the phase change material within their pore structure and limiting its migration and leakage during phase transitions [35,86]. In addition, recycled fibers and polymers may reduce the risk of cracking associated with volume changes occurring during thermal cycling [71,73].

As a result, it is possible to design multifunctional mortars that combine the required mechanical properties with enhanced energy efficiency. However, the effectiveness of PCM systems may be limited by phase change material leakage and increased production costs [86].

#### 4.4.3. Self-Healing Mortars

One of the most promising directions in the development of modern mortars is the incorporation of materials exhibiting self-healing capabilities. This process may be initiated through the use of reactive mineral additives that promote secondary hydration reactions or mineralization processes leading to the closure of microcracks [87].

The greatest potential in this regard has been reported for active mineral additives, particularly fly ash and blast furnace slag, which provide constituents participating in the continued development of hydration products [26,45,78]. Silica fume may also exert a beneficial effect by limiting the development of microcracks and promoting microstructural densification [45,46]. Recycled fibers and polymers, in turn, may reduce crack widths and support the effectiveness of self-healing processes [71,73,75].

These approaches may contribute to extending material service life and reducing the frequency of repair and maintenance operations. However, the effectiveness of self-healing is generally limited to microcracks of relatively small width [87].

## 5. Environmental Performance

Assessing the environmental performance of mortars incorporating secondary materials requires consideration of the entire life cycle of the material. In practice, particular attention is given to carbon footprint, energy consumption, demand for natural resources, and water use, as well as the benefits associated with reducing the amount of waste sent to landfill [22,29]. For construction materials incorporating recycled constituents, additional environmental benefits are associated with reduced extraction of virgin resources and the promotion of circular economy principles [1,12].

To comprehensively evaluate these aspects, Life Cycle Assessment (LCA) is widely employed as a methodology for the quantitative assessment of environmental impacts throughout all stages of a product’s life cycle, from raw material acquisition and production to use and end-of-life management [22,89].

### 5.1. Carbon Footprint

Carbon footprint is one of the most frequently evaluated environmental indicators for construction materials. In cement-based mortars, the dominant contribution to total greenhouse gas emissions is associated with the production of Portland clinker, which involves both high energy consumption and process emissions resulting from limestone decarbonization [28]. Consequently, cement production remains the primary source of environmental impact during the manufacturing stage of mortars [22].

One of the most effective strategies for reducing the carbon footprint of mortars is the partial replacement of clinker with supplementary cementitious materials (SCMs), such as fly ash, blast furnace slag, and silica fume [32]. These materials are often utilized as by-products of industrial processes, and their incorporation can reduce emissions associated with binder production while decreasing the demand for virgin raw materials [27,32].

Potentially greater emission reductions can be achieved through the use of alkali-activated materials (AAMs), including geopolymers and hybrid binders. In these systems, fly ash, blast furnace slag, and other aluminosilicate materials serve as the primary binder constituents, enabling a substantial reduction or complete replacement of Portland clinker [50]. Although numerous studies have highlighted the significant emission-reduction potential of AAM systems, the magnitude of carbon footprint reduction reported in the literature varies considerably among studies [90].

These discrepancies arise not only from differences in raw material selection, binder composition, and clinker replacement levels, but also from methodological assumptions, including system boundaries, environmental burden allocation procedures, and the consideration of transport and processing stages associated with secondary materials [89,90]. In circular solutions, the local availability of materials is also of particular importance, as it may influence both transport-related emissions and the actual environmental benefits achieved in practice. Therefore, comparisons of results reported in different studies should be made with caution and with careful consideration of the technological and methodological context of the analyses [29,90].

### 5.2. Resource Efficiency

Resource efficiency is one of the fundamental pillars of the circular economy and a key component in the sustainability assessment of construction materials [1,3]. In the case of mortars, the benefits of implementing circular solutions extend beyond reducing the quantity of waste sent to landfill and also include a reduction in the demand for virgin resources. This applies primarily to natural aggregates, raw materials used for binder production, and water resources required in technological processes [1,18].

The replacement of natural aggregates with recycled materials can contribute to reducing the exploitation of mineral resources and the environmental impacts associated with their extraction, processing, and transportation [12,33]. These benefits include reduced landscape degradation, lower energy consumption within raw material supply chains, and the extension of the life cycle of resources already introduced into the economic system [12,16,35]. However, it should be emphasized that the recovery of such materials also requires selective collection, sorting, crushing, and cleaning processes [17,91]. Consequently, the scale of the benefits achieved depends on the quality of the waste stream, the degree of material processing, and local logistical conditions [91,92]. Despite these limitations, the overall environmental balance of such solutions is often more favorable than that associated with the exclusive use of virgin materials [12,35].

A similar mechanism can be observed for supplementary cementitious materials. The use of fly ash, blast furnace slag, silica fume, and finely ground construction and demolition waste powders can reduce the consumption of limestone, clay, and other raw materials required for Portland clinker production [10,32,93,94]. Materials originating as industrial by-products are of particular importance, as their reuse increases the efficiency of resource utilization within existing industrial systems [10,72]. Nevertheless, some of these materials require additional processing, including drying, grinding, or activation, which involves additional material and energy inputs [27,50,78,95]. Consequently, actual resource efficiency depends on the balance between reductions in virgin resource consumption and the inputs required to prepare secondary materials for use in mortar production [22,27].

Water management also represents an important area for improving resource efficiency. The reuse of process water can reduce freshwater withdrawal and alleviate pressure on local water resources [65,66]. These benefits are particularly significant in regions affected by water scarcity or periodic droughts [62,63]. Furthermore, the implementation of closed-loop water systems may contribute to more efficient water use and reduce the volume of wastewater requiring further treatment or disposal [63,64]. The overall potential for water savings therefore depends on the extent to which water can be reused at successive stages of the production process [65,66].

As a result, the resource efficiency of circular solutions is derived not only from replacing virgin materials with recovered resources but also from the more effective utilization of materials already circulating within the economy and the reduction of demand for new natural resources [1,16]. However, the magnitude of these benefits depends on the degree of processing required for secondary materials, transport distances, and the technologies employed in recovery processes [22,91,92]. Consequently, the resource efficiency of individual solutions should be assessed from a systems perspective, taking into account the entire chain of material recovery, processing, and utilization [22,29].

### 5.3. Waste Diversion

Waste diversion represents one of the fundamental principles of the circular economy, as it enables the reduction of materials sent to landfill while maintaining their value through subsequent utilization cycles [1,14]. In the case of building mortars, particular importance is attached to their ability to incorporate a wide range of waste streams, including construction and demolition waste, industrial by-products, waste ceramic materials, and process water [9,10,34].

Mortars are characterized by a relatively flexible composition, making them an effective platform for the utilization of materials with diverse physical and chemical properties [23,25]. This applies to both recycled aggregates and fine-grained components used as partial cement replacements or additives modifying the properties of the composite [9,10,35]. Such an approach supports the more efficient utilization of materials already circulating within the economy and reduces the need for landfill disposal or downcycling into lower-value applications [12,36].

An important limitation is that the effectiveness of these solutions depends not only on the availability of suitable material streams but also on the performance of collection, sorting, and recovery systems. Limited recycling infrastructure, heterogeneity in local waste-management practices, and regulatory barriers may hinder the effective implementation of circular solutions in construction practice [17,96].

As a result, building mortars represent a promising platform for waste diversion strategies, enabling the incorporation of various waste-derived materials while maintaining the required performance characteristics [9,34]. However, the effectiveness of this approach depends on the efficient integration of collection, sorting, processing, and material utilization processes throughout the entire value chain [17,36,91].

### 5.4. Life Cycle Assessment (LCA)

Life Cycle Assessment (LCA) is widely used to evaluate the environmental effects of implementing circular solutions in construction materials [97,98]. This methodology enables the consideration of environmental impacts occurring throughout different stages of a material’s life cycle, from raw material extraction and production to use and end-of-life management [8,29]. Unlike assessments based on individual indicators, such as carbon footprint or resource consumption, LCA allows multiple environmental impact categories to be evaluated simultaneously [97]. Depending on the selected system boundaries, the assessment may include greenhouse gas emissions, energy and water consumption, natural resource use, waste generation, as well as impacts associated with maintenance, repair, recovery, recycling, or disposal processes [29,89].

However, the literature indicates considerable variability in LCA results reported for circular solutions [98]. These differences arise not only from the characteristics of the materials investigated but also from variations in environmental assessment scope, assumed use-phase and end-of-life scenarios, and local technological and logistical conditions [29,89]. As a result, the same solution may exhibit different levels of environmental benefit depending on the context in which it is applied, making direct comparisons among studies more challenging [22,92,98]. Despite this variability, several recurring trends can be identified in the literature, which are summarized in Table 4.

Overall, LCA studies confirm the environmental potential of circular solutions applied in building mortars, while also demonstrating that their impacts should be evaluated within the context of the entire material life cycle [22,29]. Consideration of the specific characteristics of the technologies employed, supply chains, and material sources enables a more reliable interpretation of results and facilitates the identification of the actual sources of environmental benefits and burdens [29,98].

Table 4 shows that the environmental benefits of circular material solutions are achieved primarily through the substitution of emission-intensive constituents, particularly Portland cement, supplementary cementitious materials, natural aggregates, virgin fibres, and mains water. The magnitude of the GWP reduction depends on both the type of constituent replaced and the level of substitution.

The most common strategy involves the partial replacement of Portland cement with fly ash or other waste-derived pozzolanic materials. Replacing 20–40% of Portland cement with biomass fly ash reduced GWP by approximately 11–26% [27,99], whereas the use of coal or biomass fly ash as a substitute for cement or hydrated air lime resulted in reductions of about 35–42% [22]. Waste-derived pozzolanic powders, including brick waste, glass powder, and recycled concrete powder, generally reduced GWP by approximately 15–20% at a 20% cement replacement level [30]. These results indicate that partial substitution of Portland cement typically provides a GWP reduction of approximately 10–40%, depending on the material properties, replacement ratio, and processing requirements.

The combined use of fly ash and metakaolin also reduced the demand for Portland cement. At a total replacement level of 40%, the reported GWP reduction ranged from approximately 12% to 19% [27,100]. At a 50% replacement level, substantially larger reductions of 76–109% were reported [27,100]. Values exceeding 100% indicate that the alternative mixture achieved a net-negative GWP relative to the reference mixture under the assumptions adopted in the original study. Such results are associated with specific fuel and carbon-accounting scenarios and should therefore be interpreted as scenario-dependent rather than as generally achievable reduction levels.

Substitution may also occur between different binder constituents. Replacing 30–70% of GBFS with fly ash in alkali-activated binders reduced GWP by approximately 18–65% [27,101]. In this case, the benefit does not arise from direct Portland-cement substitution, but from replacing a comparatively impact-intensive precursor with another secondary material. The reduction therefore depends on the relative environmental burdens assigned to fly ash and GBFS, as well as on activator demand and curing conditions.

Circular solutions involving aggregate substitution also show substantial potential. The production of recycled concrete aggregate may reduce GWP by up to 97% per tonne of aggregate compared with natural aggregate production [34,92]. This value applies to the aggregate-production stage and should not be interpreted as an equivalent reduction in the GWP of the complete mortar or concrete. At product level, the benefit may be lower because of crushing, sorting, transport, increased water demand, or compensatory binder use. More heterogeneous industrial waste streams replacing sand and/or cement produced lower and more variable benefits, ranging from no reduction to approximately 21% [22].

The replacement of virgin fibres with recycled polymer or textile fibres was associated with GWP reductions of approximately 25–75% [102]. These benefits are mainly related to avoiding the production of virgin polymer, glass, or mineral fibres. However, the actual reduction depends on the recycling technology, fibre dosage, and the effect of the fibres on the durability and service life of the material.

Bio-based and carbon-mineralization solutions may provide particularly high, although strongly assumption-dependent, reductions. A 30 wt.% biochar addition was associated with an approximately 86% reduction in GWP relative to the reference material [103]. This result combines the avoided use of conventional constituents with the storage of biogenic carbon in the cementitious matrix. Similarly, the use of CO_2_-treated concrete wash water and mineralized wash-water solids reduced the reported carbon impact by approximately 14% [104]. In this case, the benefit resulted from the combined replacement of mains water and part of the cement, together with permanent CO_2_ mineralization.

Overall, the reviewed solutions indicate that conventional partial substitutions of Portland cement generally reduce GWP by approximately 10–40%, while higher substitution levels, alternative binder systems, recycled fibres, and carbon-storing materials may achieve reductions of approximately 40–90% or more. The highest values are typically associated with specific carbon-accounting assumptions, avoided-production credits, or net carbon storage. Consequently, the reported percentages should be regarded as study-specific reduction potentials rather than universal performance factors, particularly because the studies differ in functional units, reference materials, system boundaries, allocation procedures, and performance requirements.

It should be noted that the environmental benefits of circular material substitutions are not limited to GWP reduction but may also extend to other LCA impact categories [22]. For mortars in which coal or biomass fly ash replaced cement or hydrated air lime, acidification potential decreased by approximately 26–37%, eutrophication potential by about 33% in the positive-impact scenarios, photochemical ozone creation potential by approximately 19–36%, and ozone depletion potential by at least 31%. Mortars containing combined industrial waste streams showed reductions of approximately 25% in acidification and eutrophication potential, 1–23% in photochemical ozone creation potential, and about 15% in abiotic depletion of elements, although some scenarios generated environmental credits exceeding the reference burdens because of the allocation assumptions applied. More modest improvements were observed for ultrafine recycled concrete powders replacing 5% of cement, for which reductions in ozone depletion, acidification, eutrophication, photochemical ozone formation, and resource depletion were generally around 3–5%, while GWP remained practically unchanged. At the same time, some variants increased fossil-resource depletion, particularly where processing and energy demand were high. These results demonstrate that the environmental performance of circular mortars should be assessed across multiple impact categories, since a reduction in climate-change impact does not necessarily prevent burden shifting to energy use or resource-depletion indicators.

A similar pattern was observed for wood bio-concretes containing metakaolin and fly ash [100]. Increasing the share of supplementary cementitious materials generally reduced several environmental impacts, but the results depended strongly on the type of additive and the energy source used for processing. Fly ash tended to perform favourably because only limited treatment, mainly drying and storage, was required, whereas metakaolin production generated additional burdens associated with kaolin extraction and calcination. In the analysed mixtures, metakaolin accounted for approximately 33–38% of ozone depletion, 13–24% of eutrophication, 21–25% of acidification, and about 95% of abiotic depletion of elements. Its contribution to photochemical ozone formation varied particularly strongly, from about 11% to 65%, depending on the calcination fuel. Wood chips were generally more advantageous for global impact categories, whereas natural gas performed better for selected local categories, including acidification and photochemical oxidation. These findings confirm that the environmental benefit of cement substitution depends not only on the replacement level, but also on the processing route and energy mix of the substitute material.

## 6. Economic Aspects

Despite the growing interest in circular solutions, their implementation in construction practice depends not only on technical and environmental performance but also on economic considerations [1,6]. The economic viability of incorporating secondary materials into mortars depends on the costs associated with raw material acquisition, processing, transportation, and quality-control requirements [12,105]. At the same time, economic benefits may arise from reduced consumption of virgin materials, lower waste management costs, and the potential extension of service life [12,106]. Consequently, the economic assessment of circular solutions requires consideration of both initial costs and the benefits achieved throughout the entire life cycle of the product [7,97].

### 6.1. Material Costs

Material costs represent one of the primary factors determining the economic feasibility of incorporating secondary materials into building mortars [1,6]. In many cases, recycled materials and industrial by-products may offer a cost-competitive alternative to conventional virgin resources, particularly when they do not require intensive processing before use [12,105]. However, these benefits are not universal, as the final cost of a material depends on its quality, degree of processing, market conditions, and logistical factors [91,96]. Consequently, the economic advantage of individual solutions may vary considerably depending on local market conditions and supply chain organization [91,105].

Supply stability also represents an important factor influencing material costs. Natural aggregates are typically extracted within established quarrying operations, enabling relatively predictable supply planning and reliable material availability for specific regions [2,107]. Similarly, cement production relies on long-term access to raw material deposits and a well-developed transport and storage infrastructure [28,108]. A different situation may arise for secondary materials, whose availability is often dependent on local economic activity and the quantity and quality of accessible waste streams [17,91]. As a result, even when such materials are available at favorable prices, limited local availability may require sourcing from more distant locations, thereby reducing the economic benefits achieved [91,105].

For aggregates, costs are closely linked to both the availability of natural resources and the efficiency of construction and demolition waste recovery processes. Recycled aggregates may reduce expenditures associated with purchasing virgin materials, particularly in regions characterized by limited access to natural aggregate deposits [12,105]. However, the overall economic balance is also influenced by selective collection, sorting, crushing, and quality-control procedures, the extent of which depends on the characteristics of the input material. Consequently, the economic viability of recycled aggregates is largely determined by the level of processing required to achieve the performance characteristics necessary for mortar applications [91].

Similar relationships can be observed for supplementary cementitious materials (SCMs). The use of SCMs, such as fly ash, blast furnace slag, and silica fume, can reduce the consumption of Portland clinker, the production of which is associated with high energy demand and increasing costs related to CO_2_ emissions [26,32]. However, the economic attractiveness of SCM utilization depends on the degree of processing required and prevailing market conditions. In many regions, these materials are no longer regarded solely as industrial by-products but have become valuable resources for the construction sector. Consequently, growing demand may increase their market value, thereby reducing their cost advantage relative to conventional materials [72,90].

Overall, the economic competitiveness of secondary materials depends less on their purchase price than on local availability, transportation distances and processing requirements.

### 6.2. Processing Costs

Processing costs represent one of the key factors affecting the economic viability of incorporating secondary materials into building mortars [12,105]. Although many waste streams may be available at relatively low cost, their utilization often requires additional preparation processes to ensure adequate quality and consistency of material properties [91].

In the case of recycled aggregates, these costs primarily include selective collection, sorting, contaminant removal, crushing, and particle-size classification [91]. The extent of the required operations depends on the characteristics of the input material and the quality requirements imposed on the final product [35,81]. In some cases, additional drying procedures or the removal of undesirable constituents may also be necessary, resulting in increased preparation costs [91,105].

Processing costs associated with supplementary cementitious materials (SCMs) differ in nature. For SCMs, ensuring adequate quality and consistency is often more important than mechanical processing alone [10,26]. This may require additional testing of chemical composition, reactivity, and variability between material batches [26,45]. For novel or non-conventional secondary materials, further verification of their influence on mortar performance may also be necessary, generating additional costs related to laboratory testing and material qualification procedures [72,88].

An important component of processing costs is also associated with the implementation of secondary materials in industrial practice [16,96]. The integration of new materials into existing production systems may require modifications to mix designs, pilot-scale trials, and additional verification of mortar performance characteristics [88]. In many cases, quality-control procedures must also be expanded to ensure the consistency of the final product [20,88]. Consequently, the total implementation costs of circular solutions include not only material preparation but also expenditures related to material evaluation, quality assurance, and integration into the production process [16,96].

As a result, the overall costs of implementing secondary materials are determined not only by acquisition costs but also by the extent of the operations required for their preparation, verification, and integration into the production process [91,105].

### 6.3. Life Cycle Costing (LCC)

Life Cycle Costing (LCC) is a tool that enables the comprehensive economic assessment of construction materials throughout their entire service life [97,109]. Unlike analyses focused solely on initial costs, this approach also considers expenditures associated with operation, maintenance, repair, and end-of-life management of materials [22,97].

For circular solutions, LCC analyses indicate that higher initial investments may be partially or fully offset by benefits arising from reduced operating costs, extended service life, or lower expenses associated with waste management [12,105]. Consequently, the economic evaluation of secondary materials should take into account not only initial costs but also the long-term benefits achieved throughout the entire life cycle of the product [29,97].

At the same time, the results of LCC analyses may vary considerably depending on the assumed service life, maintenance requirements, end-of-life management scenarios, and local economic conditions [97,105].

Moreover, as discussed in Section 6.1, the economic competitiveness of supplementary cementitious materials, including fly ash, depends on regional availability, market demand, and material prices.

Selected quantitative findings on the initial and production costs of circular material solutions used in building mortars are summarized in Table 5. These results are case-specific and should not be interpreted as comprehensive LCC assessments or evidence of long-term economic savings.

Although the available quantitative evidence primarily concerns material and production-related costs, comprehensive LCC data remain limited, particularly regarding maintenance, service life, and end-of-life costs.

### 6.4. Market Barriers

Despite the numerous environmental and economic benefits associated with the use of secondary materials, their implementation in mortar production continues to face several market barriers. Although recent standards and regulatory developments, including EN 998 [24] governing mortars and EN 197-6 [49] covering cement containing recycled construction materials, have established a framework for the incorporation of selected recycled materials into cementitious systems, important gaps remain for many emerging circular materials and mortar-specific applications. Uncertainty regarding material classification, certification procedures, long-term performance assessment, and harmonized design recommendations may reduce manufacturers’ willingness to adopt new circular solutions [8,24,49,96].

Market acceptance also represents an important challenge. Despite growing interest in materials with reduced environmental footprints, investors, designers, and contractors often remain cautious about solutions containing secondary constituents. This reluctance is primarily associated with concerns regarding quality consistency, variability among materials originating from different waste streams, and the limited availability of long-term performance data. As a result, the commercialization of many circular solutions remains slower than might be expected based on their technical and environmental performance [1,8,20,96].

The implementation of circular solutions is also influenced by regulatory and economic conditions. Increasing landfill charges, greenhouse gas emission costs, and progressively stricter environmental requirements improve the competitiveness of materials incorporating secondary resources [3,13]. At the same time, financial support mechanisms, including funding for research and development, modernization of production infrastructure, and the implementation of recovery technologies, may reduce the investment risks associated with adopting new solutions [3,113].

Consequently, the further development of the circular mortar market depends not only on technological progress but also on the evolution of regulatory frameworks, certification systems, and increased confidence among market stakeholders [8,96]. Hence, of the identified barriers, inconsistent quality of secondary materials, limited standardisation and uncertain long-term performance appear to be the most significant obstacles to wider implementation of circular mortar technology.

In general, economic viability should not be interpreted solely in terms of lower material costs. Successful implementation of closed-loop mortar technology depends on balancing material performance, environmental benefits, process requirements, and long-term economic value over the entire life cycle.

## 7. Challenges and Future Perspectives

The development of building mortars incorporating secondary materials has accelerated significantly in recent years; however, their broader implementation in construction practice continues to face substantial challenges. To date, research has focused primarily on the evaluation of technical performance and the potential environmental benefits associated with the partial or complete replacement of virgin resources with recycled materials. Considerably less attention has been devoted to long-term durability, standardization, the integration of environmental and economic assessments, and the application of digital tools supporting the design of circular materials. As a result, many promising solutions remain at the laboratory scale, and their actual application potential has not yet been fully validated. Therefore, the identification of key research gaps and future development directions represents a necessary step towards increasing the implementation of circular mortars and supporting their effective use in sustainable construction [20,96,114,115].

### 7.1. Standardization Issues

The broader implementation of circular mortars remains constrained by the insufficient level of standardization of secondary materials and technologies utilizing recycled resources [96]. Despite progress in the standardization of selected groups of construction materials, many constituents derived from industrial and construction-and-demolition waste streams are still not covered by clearly defined qualification, quality assessment, and certification procedures [49]. This limits their wider application in practice and complicates the comparison of results obtained by different research groups [20].

An additional challenge arises from the considerable variability of secondary materials resulting from their origin, usage history, and degree of processing. Even materials belonging to the same category may exhibit significant differences in chemical composition, physical properties, and reactivity, making it difficult to establish universal evaluation criteria and develop reproducible mix designs [35,91]. Consequently, manufacturers are often required to implement additional quality-control procedures, increasing the costs associated with the adoption of new solutions [91,96].

Current standardization frameworks have largely been developed for conventional materials, whereas the transition towards a circular economy requires a more flexible performance-based approach focused on material performance rather than solely on raw material composition [49,70]. Further progress in this area requires the development of standardized testing methods, qualification procedures for secondary materials, and design guidelines that enable their safe and consistent application [20]. The establishment of such frameworks represents one of the key prerequisites for increasing the use of secondary materials in building mortars and facilitating their broader implementation in industrial practice [20,96,114].

### 7.2. Long-Term Durability

Long-term durability remains one of the least investigated aspects of circular mortars. Existing studies have primarily focused on fresh-state properties, mechanical performance, and short-term durability assessments, whereas data regarding material behavior over extended service periods remain limited [20,30,35]. This is particularly relevant for mortars incorporating high proportions of secondary materials, for which the influence of aging processes on long-term performance and durability has not yet been sufficiently documented [35,116]. Recent research has shown that the durability of materials containing recycled aggregates is strongly affected by the quality and pretreatment of the recycled fraction, underlining the importance of microstructural optimization for ensuring reliable long-term performance [117].

The availability of data obtained under real service conditions is still very limited. Most existing results are derived from laboratory studies employing accelerated aging procedures, which only partially replicate the environmental conditions encountered during long-term service [20,30,34]. This limits the reliability of assessments concerning the behavior of circular mortars subjected to prolonged exposure to varying moisture, thermal, and chemical actions [34,116].

A key research priority is the development of long-term monitoring programs covering service periods exceeding 10 years, as well as predictive models capable of forecasting long-term changes in performance characteristics [20,30,34]. Expanding the database of field and long-term durability data for circular mortars is essential for their broader adoption and inclusion in design and standardization procedures [96,114], particularly in the context of developing standards addressing recycled and secondary-material-based construction products [49].

### 7.3. Integration of LCA and LCC

Despite the widespread use of Life Cycle Assessment (LCA) for evaluating circular mortars, the integration of environmental and economic aspects remains limited. Only a small proportion of available studies combine LCA with Life Cycle Costing (LCC), meaning that many proposed solutions are assessed primarily on the basis of environmental criteria alone [22,97,98]. Consequently, the actual economic feasibility of implementing circular materials, as well as the potential trade-offs between environmental benefits and economic costs, remain insufficiently understood [97,98,105].

The importance of this issue is particularly evident for materials incorporating secondary resources, whose economic performance depends on factors such as local waste availability, transportation costs, material processing requirements, quality-control procedures, and existing regulatory and fiscal instruments [8,96,105]. These factors may significantly influence the overall economic balance of a solution, even when comparable environmental benefits are achieved [98,105].

The quantitative findings summarized in Table 4 and Table 5 illustrate these relationships. Reported GWP reductions include 11–26% for biomass fly ash and 15–20% for waste-derived pozzolanic powders, while production-related cost changes range from −14.2% to +10.1% across selected mortar formulations. However, these results represent individual case studies rather than directly comparable outcomes, as they depend on functional units, system boundaries, transportation distances, processing requirements, and assessment assumptions.

A particularly important direction for future research is the development of integrated approaches combining LCA and LCC methodologies and covering the entire life cycle of mortars, from raw material acquisition to the use phase and end-of-life management [22,97,98]. There is also a need to establish harmonized assessment frameworks capable of simultaneously evaluating environmental, economic, technical, and circularity-related aspects of materials. Examples include Life Cycle Sustainability Assessment (LCSA), which combines environmental, economic, and social perspectives, as well as performance-oriented and multi-criteria assessment methods increasingly applied in sustainable construction [118,119]. Among these, the EIPI and EIPICI approaches combine ecological criteria with material-performance indicators and, in the latter case, additionally account for cost-related aspects. By integrating environmental impacts, technical performance, and economic considerations within a common evaluation framework, such methods may provide a more holistic basis for assessing circular construction materials and comparing alternative design solutions [120,121]. Although their application to mortar systems remains limited, these approaches offer considerable potential for supporting comprehensive sustainability assessments. Such integrated frameworks would enable a more reliable assessment of implementation potential and support multi-criteria decision-making in material design and selection [18,19,98].

### 7.4. Digital Tools

The transition towards circular construction is increasingly supported by digital technologies, including BIM, Digital Twins, and AI-based tools. However, their application in the field of circular mortars remains at an early stage [7,122]. This is particularly evident in the integration of material, environmental, and economic data required for the comprehensive evaluation of solutions aligned with circular economy principles [18,21].

Among the most promising approaches are Building Information Modeling (BIM) technologies and Digital Twins, which can support material information management throughout the entire life cycle of a building, facilitate the monitoring of material performance, and assist in assessing recycling potential [7,123]. At the same time, the development of digital methods, including artificial intelligence (AI) and machine learning techniques, creates opportunities for optimizing mortar formulations containing secondary materials through the analysis of large experimental datasets and the simultaneous consideration of technical, environmental, and economic requirements [11,98].

Despite rapid advances in the digitalization of the construction sector, the number of studies addressing the application of these tools to the design of circular mortars remains limited [122,123]. Future research should focus on integrating digital methods with durability assessment, LCA and LCC analyses, and material-property modeling, which may support the more effective implementation of circular solutions in construction practice [20,123].

### 7.5. Future Circular Mortars

The future development of circular mortars will be driven not only by increasing the incorporation of secondary materials but also by the design of materials with enhanced functionalities. Among the most promising directions are self-healing mortars capable of autonomously limiting microcrack propagation through autogenous healing mechanisms or the incorporation of active mineral and bio-based additives [86,124]. Such solutions may extend service life while reducing the need for repair and maintenance operations.

Increasing attention is also being devoted to smart cement-based materials whose properties can adapt to changing service conditions. Examples include systems incorporating phase change materials (PCMs), which enable thermal energy storage and contribute to improved building energy efficiency [86]. The integration of such technologies with materials containing secondary resources may expand the role of mortars beyond their traditional structural and finishing functions.

Another important area of research involves materials capable of capturing and permanently binding CO_2_. The utilization of carbonation processes and materials with enhanced carbon sequestration potential may contribute to further reductions in the carbon footprint of cement-based materials [54,56,90]. In the longer term, the combination of self-healing capabilities, intelligent responses to environmental conditions, and CO_2_ capture functions may define the next generation of circular mortars, simultaneously targeting durability, functionality, and minimized environmental impact.

## 8. Conclusions

The construction sector is under increasing pressure to reduce its environmental impact while maintaining the technical performance and economic viability of building materials. Circular material solutions for mortars represent one promising approach to addressing these challenges, involving the substitution of virgin raw materials with secondary materials and industrial by-products. This review demonstrates that the successful implementation of circular economy principles should not be viewed solely as a matter of material substitution, but rather as an integrated strategy supporting resource efficiency and reduced environmental impacts.

The analyzed studies show that the impact of circular materials on mortar performance depends primarily on the type of material, its physical and chemical properties, the degree of replacement, and the mix composition. Although many recycled materials can successfully replace conventional components without compromising key technical properties, their performance is largely dependent on the material quality and compatibility with the cement matrix. Therefore, appropriate material selection and mix-design optimization remain crucial to balancing fresh-state properties, mechanical performance, durability, and long-term functionality.

The reviewed studies indicate that the greatest environmental benefits are generally achieved when circular materials replace constituents with high embodied impacts, particularly Portland cement, virgin aggregates, and primary fibres. The scale of improvement depends on the substitution level, the environmental burden of the material being avoided, and the processing required to prepare the secondary material. In general, higher replacement levels may increase the potential environmental benefit, provided that the technical performance of the mortar is maintained.

Their benefits may also extend to other LCA impact categories, including acidification, eutrophication, ozone depletion, photochemical ozone formation, and resource depletion. However, improvements are not uniform across all categories, and additional processing, transport, or energy demand may partly offset the benefits or shift environmental burdens from one impact category to another. The results therefore depend strongly on local conditions, material-processing routes, energy sources, transport distances, system boundaries, allocation rules, and the functional unit adopted in the assessment.

Economic viability cannot be assessed solely on material costs, as processing, quality control, and long-term performance can significantly impact the overall cost-effectiveness of circular mortar technologies.

Despite significant progress achieved in recent years, a number of challenges still limit the wider implementation of circular materials in mortar production. These include variability in secondary material quality, limited standardization, insufficient long-term performance data, and the lack of integrated sustainability assessment methods.

Overall, the available literature confirms that circular materials offer significant opportunities to improve the sustainability of mortar production while maintaining the functional requirements of modern construction. However, future research should focus on integrated assessment methodologies and practical strategies supporting informed decision-making and broader adoption of circular solutions in the construction sector.

## Figures and Tables

**Table 1:** Keyword groups used in the literature search.

Theme	Keywords
Mortar	mortar, masonry mortar
Sustainability	sustainability, environmental impact, circular economy, recycling, life cycle assessment, carbon footprint, life cycle costing
Circular materials	recycled aggregates, recycled ceramic aggregates, fly ash, slag, supplementary cementitious materials, calcined clay, alkali-activated materials, geopolymers, recycled water, PET fibres, industrial waste

**Table 2:** Main advantages and limitations of recycled aggregates used in building mortars.

Aggregate Type	Main Advantage	Main Drawback
Recycled concrete aggregates (RCA)	High availability from construction and demolition waste (CDW); potential for efficient partial replacement of natural aggregates	High porosity and water absorption; presence of adhered cement paste; variable composition
Ceramic aggregates (brick and roof)	Lower density and higher porosity resulting from the firing process, contributing to improved thermal insulation	Variable mechanical performance; possible influence of glazing phases
Sanitary ceramics	High stiffness and thermal and chemical stability	Brittle behaviour; smooth/glazed surface affecting bond with cement matrix

**Table 3:** Representative supplementary cementitious materials (SCMs): typical replacement ranges and key effects on strength and durability.

SCM	Typical Replacement (%)	Strength Effect	Durability Effect
Fly ash (V) [26,32]	10–30% (~50% in specialized systems)	↓ early strength; ↑ long-term strength (due to pozzolanic reaction)	↑ resistance to chloride ingress and sulfate attack; ↓ permeability (pore refinement)
Blast furnace slag (S) [32,45]	20–60%	↓ early strength; ≈ or ↑ long-term strength (depending on activation conditions)	↑ resistance to chemical attack (e.g., sulfates); ↓ permeability; improved durability in aggressive environments
Silica fume (D) [32,46]	5–15%	↑ early and long-term strength (micro-filler and pozzolanic effects)	↓ permeability (very low permeability achieved); ↑ resistance to chloride penetration; densification of ITZ
Biomass ashes [10,47]	5–20% (highly variable)	variable (≈, ↓ or ↑ depending on composition and pozzolanic reactivity)	variable performance; potentially ↑ durability (reduced permeability) if pozzolanic; may increase porosity and reduce durability if low reactivity
CDW powders (recycled concrete (F)) [33,34,48]	5–15%	≈ or ↓ (limited reactivity; filler and packing effects)	↓ porosity (filler effect); durability strongly depends on reactivity, fineness and composition

The abbreviations V, S, D and F follow the notation used for main cement constituents in EN 197-6 [49].

**Table 4:** Reported GWP reduction potential and key influencing factors for selected circular material substitutions in building mortars.

Circular Solution	Substituted Conventional Constituent	Reported Change in GWP per Fua	Parameters Governing the Result	Ref.
Biomass fly ash replacing 20–40% of Portland cement	Portland cement	−11% to −26%	Fly-ash type and composition; replacement level; processing and drying requirements; transport distance; allocation method; mechanical performance	[27,99]
Coal or biomass fly ash replacing cement or hydrated air lime in mortar	Cement or hydrated air lime	−35% to −42%	Type of fly ash; substituted binder constituent; replacement level; processing requirements; transport distance; allocation method; superplasticizer demand	[22]
Waste-derived pozzolanic powders replacing 20% of Portland cement by volume, including brick waste, glass powder and recycled concrete powder	Portland cement	−15% to −20%	Pozzolanic reactivity; fineness; replacement level; grinding or calcination energy; electricity mix; transport distance; strength and durability	[30]
Fly ash used jointly with metakaolin to replace 40% of cement: 30% MK–10% FA and 20% MK–20% FA	Portland cement	−12% to −19%	Total replacement level; MK-to-FA ratio; calcination and grinding energy; electricity and fuel mix; transport distance; mechanical performance	[27,100]
Fly ash and metakaolin replacing 50% of Portland cement (25% MK–25% FA)	Portland cement	Approximately −88% to −111% *	Fuel used for metakaolin calcination; biogenic-carbon accounting; transport distance; allocation method	[27,100]
Fly ash replacing 30–70% of ground granulated blast-furnace slag in an alkali-activated binder	Ground granulated blast-furnace slag, GBFS	−18% to −65%	Fly-ash-to-GBFS ratio; allocation of fly ash and GBFS; transport distances; activator type and dosage; curing regime; mechanical performance	[27,101]
Recycled concrete aggregate replacing natural aggregate	Natural sand, gravel or crushed stone	Up to −97% per tonne of aggregate produced	Recycling-plant efficiency; electricity and diesel consumption; electricity mix; transport distance; aggregate quality; replacement ratio; water demand; compensatory binder dosage	[34,92]
Combined industrial waste streams, including sanitary ceramics, glass-fibre-Reinforced polymer waste, forest biomass ash and textile fibres, replacing sand and/or cement	Natural sand and/or cement	−21% to 0%	Waste-stream composition; substituted constituent; fineness; processing intensity; replacement level; transport distance; allocation method; additional binder and admixture demand	[22]
Recycled polymer or textile fibres replacing virgin fibres	Virgin polymer, glass or mineral fibres	−25% to −75%	Recycling technology; fibre dosage; waste source; transport distance; avoided virgin-fibre production; influence on service life and mortar performance	[102]
Biochar addition at 30 wt.% in a cementitious material	Cementitious constituent, depending on the investigated mix design	−86%	Biochar dosage; biomass source; pyrolysis conditions; biogenic-carbon accounting; substituted constituent; service life; end-of-life scenario	[103]
CO_2_-treated concrete wash water and mineralized wash-water solids used as mix water and partial cement replacement	Mains water and Portland cement	−14%	Cement replacement achieved by suspended solids; solids content; CO_2_ dosage and source; mineralization efficiency; treatment energy; admixture demand; avoided cement production; carbon-accounting method	[104]

* Percentage change relative to the reference formulation reported in the respective study. Negative values indicate a reduction in GWP. Results are not directly comparable across rows because the studies used different functional units, system boundaries, and methodological assumptions.

**Table 5:** Selected quantitative economic evidence for building mortars incorporating secondary and circular materials. Reported values refer to specific mortar formulations and economic assessment conditions. Economic benefit categories were interpreted according to the Life Cycle Costing approach described in ISO 15686-5:2017 [109].

Material Solution	Reference Mortar Cost	Modified Mortar Cost	Cost Change (%)	Assessment Scope and Key Assumptions	Ref.
Fly ash-based alkali-activated mortar (100% FA precursor)	50.67 USD/m^3^	43.98 USD/m^3^	−13.2%	Estimated mixture cost; transportation costs excluded; locally sourced materials assumed	[110]
Fly ash–silica fume alkali-activated mortar (90% FA, 10% SF)	50.67 USD/m^3^	46.94 USD/m^3^	−7.4%	Estimated mixture cost; transportation costs excluded; mixture composition affects economic performance	[110]
Fly ash–silica fume alkali-activated mortar (60% FA, 40% SF)	50.67 USD/m^3^	55.81 USD/m^3^	+10.1%	Estimated mixture cost; higher silica fume content increases costs under the assumed prices	[110]
Recycled concrete fine aggregates (100% replacement of natural sand)	4059.10 INR/m^3^	3481.81 INR/m^3^	−14.2%	Raw material and transportation costs; results depend on local sourcing and assumed transport distances	[111]
Recycled polyamide powder (25% aggregate replacement by volume)	88.63 EUR/m^3^	78.84 EUR/m^3^	−11.0%	Material and transportation costs; 200 km transport distance; zero purchase cost assumed for waste polyamide	[112]

Notes: FA—fly ash; SF—silica fume; INR—Indian rupee. Cost changes were calculated relative to the corresponding reference mortar as: Δ*C* = [(*C_modified_* − *C_reference_*)/*C_reference_*] × 100%. Negative values indicate cost reductions. The reported values are based on individual case studies and reflect specific mixture compositions, local market conditions, and assessment assumptions. Therefore, the results are not directly comparable across studies. The reported costs provide insights into selected aspects of life-cycle economic performance, primarily focusing on material and production-related expenditures, with the assessment scope varying across studies. Long-term maintenance costs, service life, and payback periods were not evaluated.

## Data Availability

No new data were created or analyzed in this study. Data sharing is not applicable to this article.
